# Targeted Drug Delivery Strategies for the Treatment of Hepatocellular Carcinoma

**DOI:** 10.3390/molecules29184405

**Published:** 2024-09-16

**Authors:** Yonghui Liu, Yanan Wu, Zijian Li, Dong Wan, Jie Pan

**Affiliations:** 1 School of Chemistry, Tiangong University, Tianjin 300387, China; liuyonghui@tiangong.edu.cn (Y.L.);; 2 School of Chemical Engineering and Technology, Tiangong University, Tianjin 300387, China

**Keywords:** hepatocellular carcinoma, targeting, therapy, nanocarriers

## Abstract

Hepatocellular carcinoma (HCC) ranks among the most prevalent malignant tumors, exhibiting a high incidence rate that presents a substantial threat to human health. The use of sorafenib and lenvatinib, commonly employed as single-agent targeted inhibitors, complicates the treatment process due to the absence of definitive targeting. Nevertheless, the advent of nanotechnology has injected new optimism into the domain of liver cancer therapy. Nanocarriers equipped with active targeting or passive targeting mechanisms have demonstrated the capability to deliver drugs to tumor cells with high efficiency. This approach not only facilitates precise delivery to the affected site but also enables targeted drug release, thereby enhancing therapeutic efficacy. As medical technology progresses, there is an increasing call for innovative treatment modalities, including novel chemotherapeutic agents, gene therapy, phototherapy, immunotherapy, and combinatorial treatments for HCC. These emerging therapies are anticipated to yield improved clinical outcomes for patients, while minimizing systemic toxicity and adverse effects. Consequently, the application of nanotechnology is poised to significantly improve HCC treatment. This review focused on targeted strategies for HCC and the application of nanotechnology in this area.

## 1. Introduction

Primary liver cancer (PLC) remains a major fatal threat to the safety of human life. Based on the most recent statistical data published by the International Agency for Research on Cancer (IARC) in 2022, there were approximately 860,000 new cases of primary liver cancer worldwide, and about 760,000 people unfortunately died from the disease. This has propelled liver cancer to third place in the ranking of cancer-related mortality [1], highlighting the severity of liver cancer as a public health issue. The incidence of HCC is closely related to infections with the Hepatitis B virus (HBV) and the Hepatitis C virus (HCV) [2]. Simultaneously, a variety of unhealthy dietary habits and their consequences, such as fatty liver disease, the development of alcoholic cirrhosis, smoking habits, obesity issues, diabetes, and excessive accumulation of iron in the body, contribute to the increasing risk of liver cancer [3,4]. These factors threaten a growing number of people with liver cancer. In the early stage of this disease, surgical intervention can be used to control tumor growth through liver resection or transplantation [5]. Arterial chemoembolization is the primary treatment method for patients in the middle stage [6]. For the last stage, the development of HCC can only be controlled through systemic treatment.

The liver, as one of the most vital immune organs in human bodies, not only undertakes the tasks of blood filtration and digestion but also participates in complex metabolic processes and the detoxification of toxic substances [7]. Due to the importance of its multiple roles, HCC often encounters challenges from the complex tumor microenvironment during the treatment process. The treatment of HCC needs to confront the complex tumor microenvironment, which includes a weakly acidic environment with low pH, excessive aggregation of M2 tumor-associated macrophages, hypoxia, abundant blood supply, a high tendency for vascular invasion and metastasis, high chemokine expression, overexpression of enzymes, high levels of redox reactions, strong immunosuppression, etc., [8,9,10,11]. The combined effect of these adverse factors results in limited treatment efficacy for liver cancer, making it prone to recurrence and accompanied by a high risk of mortality.

Chemotherapy plays an indispensable role in the treatment of liver cancer; however, traditional chemotherapeutic drugs are not effective in achieving good control over the tumor. Their efficacy is limited by multiple challenges, including insufficient selectivity for liver cancer tissues, which makes it difficult for the drugs to precisely target the lesions. The short residence time of the drugs in the body causes their therapeutic effects to fail to exert fully. The significant systemic toxic reactions increase the treatment burden on patients. The ease of inducing multidrug resistance further weakens their long-term therapeutic efficacy. These factors collectively lead to the clinical efficacy of the drugs falling short of the ideal state [12,13]. Sorafenib is a multi-target tyrosine kinase inhibitor (TKI) [14,15,16]. In the past decade, it has remained the first-line treatment for advanced HCC [17,18]. After 2017, the approval of new drugs like refametinib for the treatment of HCC has changed this situation [19]. These have shown better therapeutic effects than previous medications in clinical treatment (Table 1). Notwithstanding, some issues that exist with traditional chemotherapeutic drugs, such as multidrug resistance and significant side effects, have not been fundamentally resolved [20,21].

To address these issues, scientists have turned their attention to nanotechnology. The introduction of nanotechnology into chemotherapy can achieve precise delivery and controlled release of drugs, enhancing treatment efficacy and accuracy in targeting the lesioned area. Concurrently, this approach substantially diminishes the toxic and side effects of chemotherapy on healthy tissues, offering a safer and more effective therapeutic option for liver cancer patients. Nanoparticles, with their large specific surface area and high stability, are emerging as the preferred candidates for drug delivery systems, optimizing drug absorption and release efficiency. Nanomedicines, in contrast to conventional drugs, protect the therapeutic payload prior to administration [23]. The higher drug-loading capacity allows them to carry more drug molecules, and they possess a high degree of targeting specificity and accuracy. They can perform precisely on epithelial tissues, promoting full absorption of the drug, and altering the pharmacokinetics and distribution characteristics of the drug. At the same time, this technology effectively prevents the premature degradation of chemotherapy drugs in the bloodstream, significantly improving the pharmacokinetic behavior and in vivo distribution. It can significantly enhance the intracellular concentration of anticancer drugs to optimal levels within a short period, minimizing the side effects associated with cytotoxic agents. Therefore, the application of nanotechnology in chemotherapy has become a focal point and cutting-edge field in current medical research, demonstrating immense potential for development and broad prospects for application [24,25]. Above all, this review dug into the cutting-edge field of nanotechnology and elaborated novel targeting strategies for HCC therapy. Based on common targeting approaches, carbohydrate receptor targeting, precise targeting mechanisms about secreted proteins, and tumor vascular networks were further introduced. In addition, it also described the application of these advanced nanomedicines in the treatment of HCC with a variety of therapies including chemotherapy, phototherapy, and immunotherapy. The inventive usage of nanotechnology points the way to the development of HCC treatments and is an important innovation in existing treatments.

## 2. Targeting Strategies for HCC

**Scheme 1:**
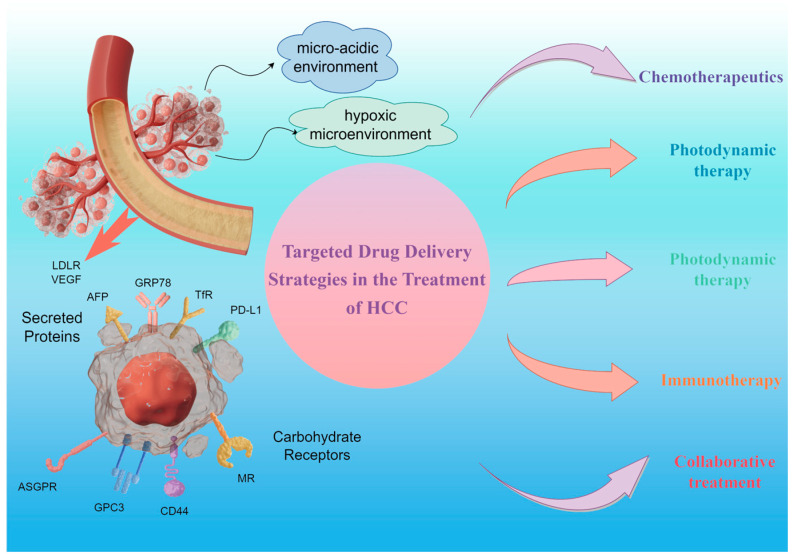
Targeted strategies for treating HCC. This diagram was drawn in Figdraw.

A nanodrug delivery system (NDDS) is an ideal material that can be used in the treatment of liver diseases. However, it is undeniable that the liver has a strong shielding function against foreign substances, meaning the nanomedicine will eventually be deposited and metabolized in the liver or spleen, which will reduce the bioavailability of the drug, make the therapeutic effect of the drug worse, and even cause hepatotoxicity, leading to a series of toxic side effects [26]. Nanoparticles are taken up by the reticuloendothelial phagocytosis system (RES) in the liver, which consists of Kupffer cells [27]. Once nanoparticles enter the circulation, they first bind to plasma proteins, a process that promotes nanoparticle opsonization, so that the surface properties of the nanoparticles are altered to make them more easily recognizable. Subsequently, the opsonized nanoparticles trigger endocytosis by macrophages, a key component of the RES responsible for capturing and depositing these nanoparticles in the liver. This process effectively prevents the re-entry of nanoparticles into the blood circulation and ensures their efficient accumulation and processing in the liver [28]. According to experiments, negatively charged nanoparticles are more likely to be absorbed by the RES and cause hepatotoxicity; therefore, hydrophilic groups are usually encapsulated in the periphery of the formed NDDS to mask the charge to mitigate the clearance of the drug by the liver [29,30]. On the other hand, the efficiency of nanoparticles in reaching the site of action is deeply influenced by their size, shape, and other factors. In the field of targeted cancer therapy, nanomedicines have demonstrated a number of compelling advantages due to their unique EPR (Enhanced Permeation and Retention) effect [31,32,33]. The core of this effect stems from the anatomical complexity and pathophysiological abnormalities specific to the tumor vascular network [34,35]. Under the merged influence of these factors, the high selectivity and concentrated distribution of macromolecular drugs within tumor tissues are significantly enhanced [36]. This mechanism ensures that, after systemic administration, the macromolecular drugs can preferentially penetrate and remain inside the solid tumor for an extended period, thus achieving precise delivery and sustained action of the therapeutic drugs, and greatly improving the precision and efficiency of the treatment. The efficacy of this impact has been fully demonstrated in numerous animal experiments, and its clinical value has also been verified in human patients, including in the treatment of malignant tumors such as liver cancer, renal cancer, and metastatic breast cancer, where it has shown encouraging therapeutic prospects [37,38,39]. This marks the EPR effect as a key mechanism in the NDDS, which opens up new avenues for cancer therapy and greatly enhances the targeting and efficiency of treatment (Scheme 1).

### 2.1. Active Targeting 

Liver tumor tissue exhibits a characteristic tumor microenvironment configuration, which is shaped by a diverse cellular population including, but not limited to, aneuploid cancer cells, stromal cells, deeply infiltrating immune cells, and a wide range of other bioactive factors involved in regulation [40]. Therefore, a microenvironment-responsive nano-drug delivery system can be designed by using the specific tumor microenvironment of hepatocellular carcinoma to precisely deliver the drugs into hepatocellular carcinoma tissues, hepatocellular carcinoma cells, mesenchymal stromal cells or subcellular organelles, so as to achieve the precise release of the drugs; through this targeted release mechanism, the effective concentration of the drugs in the focal areas of the liver cancer can be significantly increased, which can greatly enhance the therapeutic effect of the liver cancer, and thus open a more efficient and precise pathway for hepatocellular carcinoma treatment. Through this targeted release mechanism, the effective concentration of the drug in the focal area can be significantly increased, thus greatly enhancing the therapeutic effect on liver cancer and opening up a more efficient and precise way for liver cancer treatment.

#### 2.1.1. Targeting Tumors with Secreted Proteins

**Figure 1:**
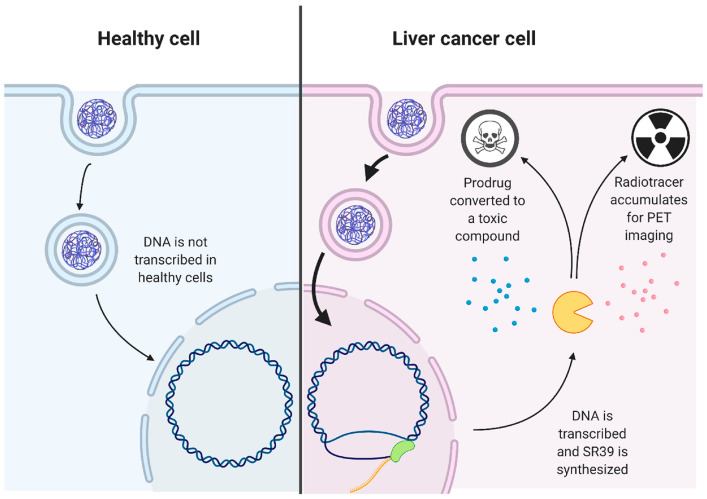
Treatment of HCC by targeting AFP [42]. Reprinted with permission from [42]. Copyright 2022, The American Association for the Advancement of Science.

Alpha-fetoprotein (AFP) is a major biomarker of hepatocellular carcinoma and is found in low levels in normal tissues, where its expression is difficult to detect. However, in up to about 80% of primary HCC cases, the level of AFP is significantly elevated, making it an important diagnostic marker in these cases [41]. In response to this phenomenon, Vaughan et al. designed poly-amino ester (PBAE) nanoparticles that are capable of delivering simplex virus type 1 sr39 thymidine kinase (sr39) DNA to human HCC cells, and facilitate the phosphorylation of the prodrug ganciclovir (GCV), transforming it into a potent nucleotide analog that efficiently halts DNA polymerization in actively dividing cells, thereby achieving safe and effective tumor control. Experiments have shown that these nanoparticles can reduce tumor size to 62%, achieving effective targeted therapy for HCC (Figure 1) [42].

There is a GRP78 protein in the HCC cell membrane that could be a target for HCC therapy [43]. GRP78, a member of the heat shock protein 70 (Hsp70) family [44], is a chaperone protein located in the endoplasmic reticulum (ER). Its primary role is to facilitate the correct folding and efficient transport of proteins [45]. Moreover, GRP78 is considered a key marker of the endoplasmic reticulum stress state [46]. The expression level of GRP78 is significantly upregulated under chronic stress conditions, a phenomenon that contributes to the escape of tumor cells from the immune surveillance system of the body and represents a more aggressive phase of tumor malignancy. Jiang and colleagues have designed and developed a nanocage structure, the GRP78-targeted HccFn-DOX system, which cleverly utilizes a Pyrococcus-derived ferritin nanocage (HccFn) as a carrier with high stability. The SP94 peptide in this system is exposed on the external side of the ferritin (from Pyrococcus) nanocage (HccFn), with the chemotherapeutic agent DOX encapsulated within the HccFn cavity. The SP94 peptide can bind specifically to HCC cells [47], and subsequently serve as a ligand for a targeted therapeutic approach against HCC [43,48,49]. The findings revealed that the fabricated HccFn-Dox nanocages demonstrated potent therapeutic efficacy against HCC, concurrently reducing the toxic side effects of the drug, thus offering an innovative and serviceable strategy for the treatment of HCC.

The transferrin receptor (TfR) is highly expressed in numerous tumor cells including HCC cells due to abnormal iron metabolism [50,51,52,53,54]. Consequently, it can serve as a targeted approach for the selective treatment of HCC [55]. Transferrin (Tf) is a protein present endogenously for the efficient transport of iron into the cells that have overexpressed TfR [56]. Given its unique properties, Tf has been extensively researched and applied across various fields, including by serving as a specific delivery vector for diverse drug molecules [57,58]. As a targeting ligand for a wide range of nano-formulations, Tf helps to achieve more precise and efficient delivery of drugs to therapeutic targets [59,60,61]. Applying this property, Wei and others developed transferrin-modified polycarbonate-based doxorubicin (Tf-Ps-DOX) [62]. This carrier adeptly utilizes the TfR-mediated mechanism to facilitate efficient endocytosis by tumor cells. The drug delivery system not only demonstrates exceptional stability, ensuring the integrity and efficacy of the drug during transit, but also exhibits high tolerance, reducing adverse effects on patients. Additionally, it is characterized by its ability to intelligently respond to specific environments for drug release. This Tf-guided polymer-encapsulated doxorubicin strategy reveals great potential and attraction for chemotherapeutic applications targeting hepatocellular carcinoma. The drug can be highly specifically and precisely recognized by and attach to transferrin receptors on the surface of cancer cells for targeted drug delivery. This property suggests that it has great potential to enhance the efficacy of HCC treatment while significantly reducing the side effects on non-cancerous cells, creating an innovative and effective therapeutic pathway in the field of hepatocellular carcinoma treatment.

**Figure 2:**
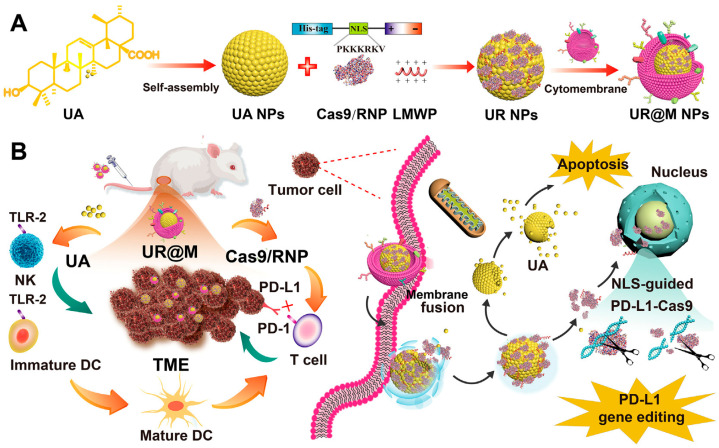
The mechanism of action of UR@M NPs for HCC immunotherapy. (**A**) Engineering of bionic UR@M NPs incorporating UA and CRISPR technologies. (**B**) UA combined with PD-L1 gene therapy to enhance immunotherapy with nanomedicines [67]. Reprinted with permission from [67]. Copyright 2024, Elsevier.

Immunotherapy has become one of the most crucial methods for cancer treatment, which is gaining more and more attention and has gradually become one of the most powerful means of treating terminal cancers [63,64]. The CRISPR/Cas9 gene editing technology enables disruption of immune checkpoint gene loci, thereby reducing the expression of PD-1/PD-L1, which helps to decrease the proliferation and spread of cancer cells in patients with end-stage HCC [65,66]. Zhang and colleagues ingeniously devised a CRISPR/Cas9 gene-editing system targeting PD-L1, utilizing a universal triterpene template and ursolic acid (UA), a potent anticancer agent, and cleverly fused this system with UA self-assembly technology to prepare a membrane-encapsulated biomimetic nano-delivery platform called UR@M, which has good stability and dispersibility (Figure 2) [67]. UA has significant anticancer activity and, together with the Cas9/sg-PD-L1 ribonucleoprotein complex (RNP) and a permeabilizing peptide (low molecular weight fisetin, LMWP), forms nanodrugs by self-assembly for the therapeutic approach to HCC. UA is distinguished by its dual functionality, effectively curbing the proliferation of cancer cells while also possessing the remarkable ability to activate the immune system [68,69]. As a result, the prepared nanomedicine UR@M exhibited remarkable stability and strong protective efficacy against sgRNA, a property that enabled it to significantly inhibit the proliferation and regulate the apoptotic process of HCC cells, while inducing a highly efficient editing ability against the PD-L1 gene. The synergistic immunotherapeutic effect of UR@M leverages a dual mechanism to achieve the treatment of HCC. It activates the innate immune pathway via the TLR-2-MyD88-TRAF6 signaling cascade and simultaneously targets the knockdown of the PD-L1 gene, thereby enhancing the immune response of body.

#### 2.1.2. Targeting Tumors with Carbohydrate Receptors

Carbohydrates serve as essential media for energy storage and transport within the human body, and are also vital molecular components in the domain of bioinformatics, where they assume a pivotal role in numerous critical physiological processes. These carbohydrate molecules are able to accurately regulate the flow and transmission of biological information under their highly specific recognition of biomolecules such as proteins, thus ensuring the smooth functioning of the complex and fine-grained regulatory network within the living organism [70]. On the surface of malignant tumors, there exist many cancer markers adhering to glycans, which can act as receptors on the surface of cancer cells. At the same time, carbohydrate molecules themselves are characterized by low toxicity and good biocompatibility, which has led to an increasing interest in them in the field of cancer therapy [71]. Currently, many researchers have synthesized well-targeted, highly transfected, and low-toxicity carbohydrate-targeting drug vectors, marking significant advancements in the realm of cancer treatment [72].

**Figure 3:**
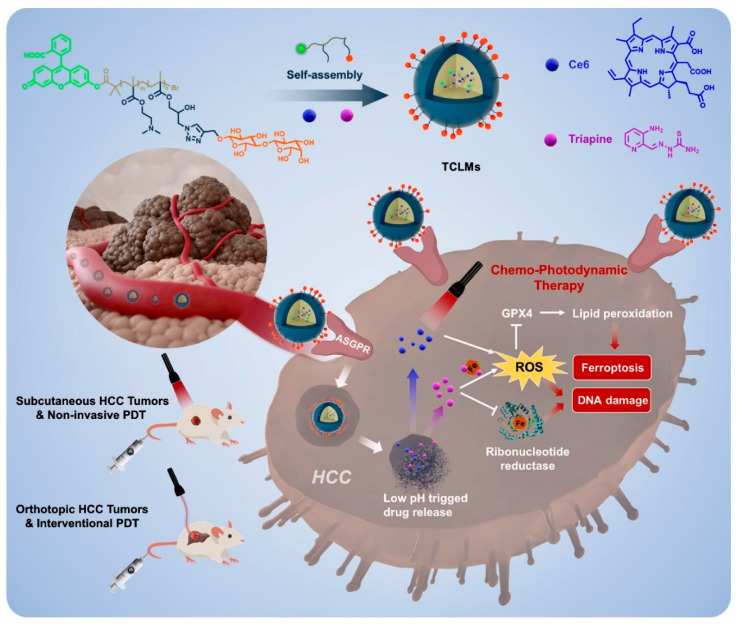
Construction strategy of TCLMs and schematic diagram of target-generating chemo-photodynamic therapy [76]. Reprinted with permission from [76]. Copyright 2021, Elsevier.

ASGPR, a highly efficient endocytosis receptor with a unique affinity for lactose, is typically found in high concentrations on the surfaces of liver parenchymal cells and hepatocellular carcinoma cells in dairy animals. It is integral to the endocytosis process, specifically recognizing, binding to, and internalizing carbohydrates from the circulating bloodstream [73]. It can specifically contain oligosaccharides or glycoproteins of D-galactose and N-acetyl-D-galactosamine and actively translocate them to the interior of the hepatocyte where they are metabolized [74,75]. Therefore, its recognition of glucose and galactose can be exploited to link the drug to carbohydrates, resulting in better targeting of the drug, thus increasing the efficacy of the drug and lessening adverse effects. Capitalizing on this attribute, Zhang and his team designed and contrived an innovative drug delivery system based on nano microspheres, which were loaded with Triapine and the photosensitizer Ce6, to form a nano cellular drug delivery system TCLMs with good stability (Figure 3) [76]. Triapine is a compound that sequesters iron, characterized by a thiosemicarbazone (TSC) structural framework [77]. It induces apoptosis in cancer cells by inhibiting key iron-containing enzymes like ribonucleotide reductase (RR), which plays a significant role in the synthesis and repair of DNA [78,79]. Furthermore, Triapine can form hydroxyl radicals with iron via the Fenton reaction, forming lipid peroxide (LPO) deposits that evoke ferroptosis [80]. The photosensitizer Ce6 promotes ferroptosis in tumor cells by generating unilinear oxygen, depleting intracellular glutathione (GSH), and reducing glutathione peroxidase 4 (GPX4) [81,82]. Modification of lactose on the surface of TCLMs enables its targeting of HCC cells, allowing drug release in the tumor microenvironment and combined chemo- and photodynamic therapy of tumors, showing the encouraging potential of polysaccharide-modified nanomedicine delivery systems for the combined treatment of HCC.

GPC3, a heparan sulfate proteoglycan, is critically involved in regulating the advancement of HCC. It is stably anchored to the cell membrane and is found to be overexpressed in 70% of HCC tumors, while it is absent in normal adult liver tissue [83]. In recent years, GPC3 has emerged as a potential target for molecular imaging and has also been widely explored for therapeutic intervention in HCC [84,85,86]. Deng et al. have engineered an NDDS for HCC by conjugating GPC3-targeting enzymes with Fe_3_O_4_–glucose oxidase to create Fe_3_O_4_–glucose oxidase (GOD) ––GPC3 nanoparticle peptides (FGP NPs). In vitro stability experiments showed good stability of this NDDS. These nanoparticles facilitated integrated ultrasound/photo-acoustic therapies within the intricate hepatic microenvironment [87]. Among them, Fe_3_O_4_ can produce Fenton-like reactions in the tumor microenvironment, generating reactive hydroxyl radicals and oxygen species [88]. GOD catalyzed the production of H_2_O_2_ from glucose, which reacted with Fe_3_O_4_ to produce hydroxyl radicals with strong oxidizing properties that kill tumor cells [89]. The nanoparticles are also useful for tumor imaging, helping to improve the accuracy of small liver cancer detection. FGP NPs offer innovative approaches for the treatment of HCC, as well as potentially effective options in the field of tumor imaging.

Extensive prior research and experimentation have consistently shown that the cluster of differentiation 44 antigen (CD44) is linked to the advancement of HCC [90]. CD44 is a cell surface glycoprotein receptor that carries the burden of regulating many biological functions in the organism [91,92], and it is implicated in the nuanced regulation of hematopoietic processes and exerts influence over the activation status of lymphocytes, facilitating the recirculation and homing of lymphocytes throughout the body. Within the realm of tumor biology, CD44 is closely associated with the progression and metastatic potential of cancer cells [90,93,94]. Experiments have proved that the concentration of CD44 in HCC patients is significantly higher than that in cirrhotic patients. In the field of tumor therapy, hyaluronic acid (HA) can be applied in combination with CD44 for drug targeting. The combination of CD44 with HA has also been confirmed to regulate a spectrum of phenotypic traits in cancer cells, such as tumor progression, metastasis, and proliferation [95]. Through this property, Zhang et al. designed Janus magnetic mesoporous silica nanoparticles (MSNs), HA-MSN@DB, that can be loaded with both DOX and berberine (BER), which enabled drug access to tumor tissues through CD44 receptor-mediated targeting while remaining stable in PBS [96]. These MSNs could assume a therapeutic function in HCC by dual-modal action, offering magnetically targeted chemotherapy and magnetic resonance imaging (MRI)-guided, heat-enhanced gene therapy [97,98,99]. BER inhibited the Caspase-3-iPLA2-COX-2 pathway in the organism, reduced DOX-induced HCC repopulation, and integrated DOX and BER for effective HCC chemotherapy without relapse [100,101,102]. Validation of the nanocarriers by multiple characterization experiments demonstrated that the nanocarriers enable safe and effective drug delivery, providing a basis for the future design of HA-functionalized Janus nanoparticles for use in anti-relapse strategies for HCC.

The mannose receptor (MR) is an important endotoxin receptor whose functions include scavenging endogenous molecules, facilitating antigen presentation, regulating cellular activation, and trafficking, and has also been implicated in immune escape and metastasis of tumors [70]. MR is abundant within the cells of the liver, encompassing tumor cells, dendritic cells, macrophages, as well as a variety of nonparenchymal liver cells, such as hepatic endothelial cells and Kupffer cells [103,104]. Macromolecules such as glycoproteins can enter the cell through endocytosis by binding to mannose residues [105]. Xiao et al. combined thermal ablation with immune checkpoint blockade (ICB) to construct antigen-capturing stable nanoplatforms modified with mannose as a targeting ligand on their surfaces [106]. This platform enables the co-delivery of tumor-associated antigen (TAA) and N6-methyladenosine demethylase (m6A) inhibitors (i.e., fat mass and obesity-associated gene (FTO) inhibitors) into tumor-infiltrating dendritic cells (TIDCs) to more effectively suppress the metastasis and recurrence of HCC following thermal ablation. They encapsulated the FTO inhibitor (FB 23-2) encapsulated within the pores of mesoporous polydopamine (MPDA) nanoparticles, while maleimide (mal) was utilized as an antigen-capturing agent and mannose was employed as a dendritic cell-targeting ligand. These components were surface-modified onto the MPDA nanoparticles via a polyethylene glycol (PEG) spacer, culminating in the creation of a sophisticated nanomedicine. When the drug enters the tumor tissue by injection, the TAAs released after thermal ablation of the tumor are captured by reacting with methylation-modifying groups and are subsequently endocytosed by TIDCs through mannose-mediated targeting, enabling ICB therapy [107,108]. The results suggest that co-delivery of TAA and FTO inhibitors to TIDCs is anticipated to trigger a robust anti-tumor immune response by enhancing m6A methylation, fostering DC maturation, and activating anti-tumor immunity following the thermal ablation of HCC, thereby assisting in the induction of ICB to inhibit distant tumor growth and metastasis.

#### 2.1.3. Targeting Tumors with the Vasculature

The growth of HCC is characterized by genetic mutations in hepatocytes, a process that triggers an uncontrolled state of cell growth and proliferation. Given the unique physiological environment of liver tissue, the tumor microenvironment formed by HCC in the liver is significantly different from that of other tumor types and exhibits characteristic pathological features. In particular, HCC, as a highly vascularized tumor type, suffers from the overexpression of a large number of growth-promoting factors, which further promote tumor growth and proliferation. Based on this feature, the treatment of HCC can be achieved by inhibiting production of tumor growth-promoting factors. Effective intervention in tumor growth can be achieved by precisely targeted delivery of drugs to the vascular region of the tumor using nanotechnology. This therapeutic approach can directly act on the vascular system of the tumor, thus cutting off its source of nutrients and inhibiting its further development and spread. The inhibition of angiogenesis not only opens up new avenues for the treatment of HCC, but is also regarded as a promising new approach in the field of cancer therapy, which is expected to significantly improve the survival rate and quality of life of cancer patients in the future [109].

**Figure 4:**
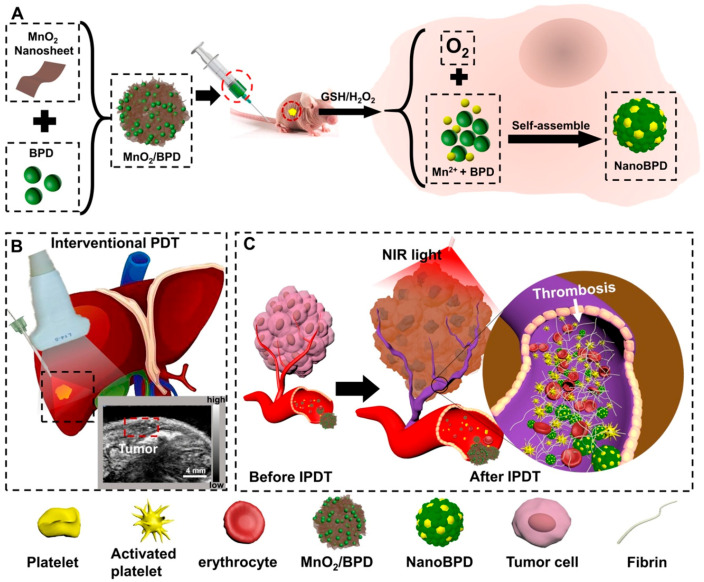
Schematic representation of MnO_2_/BPD synthesis and intervention in PDT-induced thrombosis. (**A**) After synthesized in vitro, the MnO_2_/BPD nanoparticle was injected to undergo the reduction and self-assemble in vivo. (**B**) Schematic representation of IPDT under the guidance of ultrasound [113]. Reprinted with permission from [113]. Copyright 2020, American Chemical Society.

Verteporfin (a benzoporphyrin derivative [BPD]) is a clinically used photosensitizer. Its unique feature is that it can bind specifically to the low-density lipoprotein receptor (LDLR), which enables precise targeting of tumor vascular endothelial cells (TVEC) [110,111,112]. Wang et al. prepared MnO_2_/BPD nanocomposites using manganese dioxide (MnO_2_) nanosheets as a carrier for binding verteporfin to tumor vascular endothelial cells (TVECs) (Figure 4) [113]. The MnO_2_ nanosheets, serving as the carrier, enhanced the targeted accumulation of the drug. Once endocytosed, MnO_2_ underwent a reduction reaction in the presence of high intracellular concentrations of glutathione (GSH) or hydrogen peroxide (H_2_O_2_) and was converted to Mn^2+^. The subsequent self-assembly of Mn^2+^ with the porphyrin ring and carboxylate groups of BPDs gave rise to nanoBPDs. These nanomaterials induced apoptosis and initiated a coagulation cascade response in TVECs, while simultaneously visualizing tumor vascular density. The stability of the nanocomposites was confirmed by UV–Vis spectroscopy; they did not aggregate or precipitate in serum for 72 h. This dual functionality allowed for the eradication of tumors and the monitoring of drug efficacy, offering a novel therapeutic option for patients with unresectable HCC.

Vascular endothelial growth factor (VEGF) is an important growth factor secreted in tumor cells and plays an important role in tumor angiogenesis [114]. Many scientists are targeting VEGF and its receptors to develop drugs that can inhibit the development of HCC through anti-tumor angiogenesis, such as the first-line therapeutic agent sorafenib [115]. Therefore, the treatment of HCC can be achieved by nanotechnology, targeting VEGF. The miR-128-3p Agomir (NA-miR-128-3p), a nanoparticle delivery system that enables slow drug release, was developed by Wang et al. [116]. The nanoparticles were prepared from pentasodium tripolyphosphate (TPP) as a cross-linking agent in combination with chitosan hydrochloride and miR-128-3p Agomir, and the target aptamer was encapsulated within chitosan nanoparticles via a self-assembly process. The system has the potential to disrupt the progression of HCC by modulating the expression of AKT1. microRNAs (miRNAs) are short RNA molecules that target different molecules in different signaling pathways to achieve the regulation of the pathways [117], and they are the key regulators in HCC [118]. However, miRNAs are extremely unstable and are easily broken down and metabolized by RNA enzymes in serum and other body fluids [119]. However, miRNAs can be modified using Agomirs to improve serum stability, increase cellular uptake, and improve intracellular stability [120]. In addition, studies proposed a novel, efficient, long-lasting, multi-mechanism, low-dose co-administration strategy and showed significant anti-tumor effects. NA-miR-128-3p can be combined with orexin B to significantly affect HCC progression by interfering with the VEGF and PI3K-AKT pathways [121,122], which is a new co-administration strategy and provides a new idea for HCC treatment. 

In recent years, the therapeutic field for intermediate and advanced HCC has witnessed remarkable breakthroughs, with the rapid rise of targeted molecular therapies represented by multi-targeted tyrosine kinase inhibitors (TKIs) and anti-angiogenic antibodies, which have become important milestones in the treatment of the disease. Sorafenib and lenvatinib, as outstanding representatives of this approach, have become classic drugs in this field [123,124]. However, oral TKIs often have to overcome a series of biological barrier challenges before reaching the site of action, which directly affects the stability and bioavailability of the drug. Liu et al. developed a supramolecular tumor-associated macrophage (TAM) reprogramming poly-TLR 7/8a nanomodulator (p(Man-IMDQ)NRs) co-assembled with lenvatinib-loaded nanomedicines (PCN-Len nanoparticles, NPs) and oxidized dextran (DX) and loaded with. This supramolecular hydrogel drug delivery system (PLDX-PMI) could target the abnormal vascular network in the tumor microenvironment of HCC in situ [125]. As an anti-angiogenic agent, PCN-Len NPs targeted tyrosine kinases in vascular endothelial cells, leading to the downregulation of VEGF-A and Ang-2 expression, disrupting tumor angiogenesis subsequently decreasing the microvessel density [124,126,127,128]. p(Man-IMDQ) NRs reduced pro-angiogenic M2-type TAM via mannose receptors binding to anti-angiogenic M1-type TAM, reduced VEGF secretion [129], and further inhibited vascular endothelial cell migration and proliferation [130,131]. Overall, the findings in this work highlight the importance of TAM reprogramming in augmenting anti-angiogenic therapy for in situ HCC and in providing a synergistic approach to tumor therapy with advanced hydrogel-based delivery systems.

### 2.2. Tumor Microenvironment Stimulus Response Targeting

In the tumor microenvironment of HCC, there are a series of biochemical changes that are different from those of other normal tissues, which are notable in terms of deviations in pH, abnormalities in redox potential, increased hypoxia, elevated local temperatures, and altered patterns of expression of specific enzymes and proteins, which are accompanied by significant differences in stromal cellular composition and content [10]. Together, these complex microenvironmental features shape a unique ecosystem that favors tumor growth, invasion, and metastasis [132]. Targeted drug release of nanoparticles to tumor tissue can therefore be achieved by external stimulation through these differences.

#### 2.2.1. pH-Responsive Drug Release

pH stimulus responsiveness is a widely studied response to an exogenous stimulus, and the Watt effect causes an increase in lactate production resulting in a micro-acidic environment in tumor tissue [133,134]. Based on this, a series of pH-responsive nanodrug delivery systems have been developed. Wu et al. developed a stable biomimetic nanodrug delivery platform, LT@PAE@CCM, which consists of a lenvatinib core encapsulated with a pH-sensitive polymer, i.e., poly(β-amino ester)-poly(ethylene glycol)-amine (PAE-PEG-NH2), and a shell formed by a cancer cell membrane (CCM) [135]. The material effectively targets liver cancer cells and delivers LT to the tumor site, significantly reducing tumor size [136]. The experimental results show that this nanoplatform has a high drug-loading capacity, enables tumor-specific targeting and precise drug release, and has good biocompatibility and anti-tumor effects. It provides a new strategy for the precise treatment of tumors with first-line clinical drugs.

#### 2.2.2. Environmentally Responsive Drug Release in Hypoxia

HCC creates a hypoxic microenvironment in the tumor due to its lack of abundant vascular environment and aberrant proliferation [137,138], a property that provides a new strategy for drug delivery, i.e., designing drugs capable of targeting this hypoxic environment to achieve highly effective targeted therapies in the peripheral region of liver tumors. Shi et al. designed and fabricated a nano vaccine capable of enhancing the killing of hepatocellular carcinoma cells in a hypoxic environment while improving the immunogenic tumor microenvironment [139]. The vaccine is loaded with a zeolite imidazolium ester backbone of the hypoxia-activated prodrug tirazamine (TPZ) and the immunoadjuvant laquinimod, which triggers a strong anti-tumor immune response by enhancing the influx of immune cells, particularly cytotoxic T cells, into both the primary and distal regions of the tumor. TPZ, a hypoxia-activated prodrug, is metabolized by intracellular reductases to generate highly reactive free radicals, which in the hypoxic environment induce DNA strand breaks and increase toxic effects on topoisomerase II [140]. It has been shown that TPZ improves tumor microcirculation by inducing cell cycle arrest and downregulating the expression of hypoxia-inducible factor-1α (HIF-1α) and VEGF [141,142]. In studies, TPZ and R848 (TLR 7/8 dual agonist) were encapsulated in a backbone of zeolite imidazolium ester backbone-8 nanoparticles [143,144], which were microloaded by porous gelatin, and, together, they constituted the nano vaccine, TRZM, to achieve efficient inhibition of primary tumor growth and tumor metastasis. The construction of this nano vaccine provides a platform that enables the attainment of elevated drug concentrations within tumors, robust anti-tumor efficacy, and minimized systemic toxicity.

## 3. Targeted Therapies for HCC

### 3.1. Chemotherapeutics

In recent years, small molecule drug chemotherapy has become one of the effective modalities for the treatment of HCC and has made remarkable progress. A variety of small molecule chemotherapeutic drugs for HCC have entered the clinical trial stage one after another, providing new treatment options for patients. The introduction of nanotechnology to improve and optimize chemotherapeutic drugs not only significantly improves the therapeutic efficiency of the drugs and enables them to target cancer cells more precisely, but also greatly reduces the side effects of traditional chemotherapy, bringing HCC patients safer and more effective treatment options. Liu et al. developed a drug nanocarrier for targeted chemotherapy of liver cancer [145]. They wrapped HepG2 cell membranes around poly(lactic-co-ethanolic acid) (PLGA) nanoparticles to create the nanocarrier HepM-PLGA, which was loaded with the conventional chemotherapeutic drug DOX. Surface protein interactions on the membrane of homotypic cancer cells endowed the nanocarrier with an excellent homotypic targeting ability and significant immunocompatibility, which could effectively prolong the circulation time of the drug in vivo and improve the drug delivery efficiency [146,147]. These nanocarriers can effectively extend the drug circulation duration and enhance the drug delivery efficiency throughout the body [148,149,150,151,152]. Experiments showed that HepM-PLGA had excellent targeting of HepG2 cells, carrying DOX directly to the tumor area and significantly reducing the tumor volume. Therefore, the biomimetic HepM-PLGA platform is considered as a promising and potent nanoplatform for liver cancer chemotherapy and provides new strategies for the development of optimal drug delivery platforms for other cancers, offering new perspectives for future tumor chemotherapy.

### 3.2. Photodynamic Therapy

**Figure 5:**
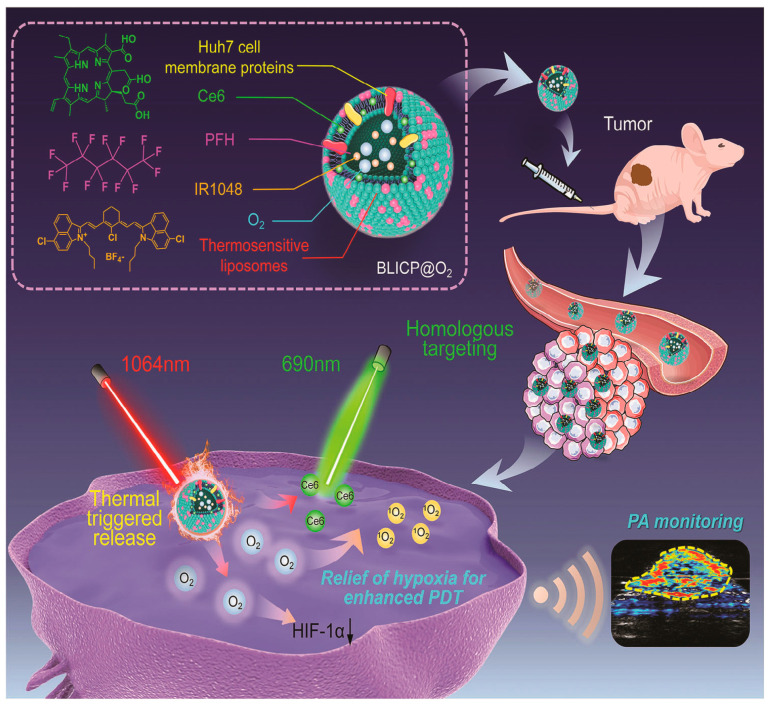
NIR-II photoacoustically guided controlled oxygenation bio-nanoparticles for highly specific HCC photodynamic therapy [158]. Reprinted with permission from [158]. Copyright 2024, Wiley.

Photodynamic therapy (PDT) is an emerging therapy for tumor treatment, which is a non-invasive anti-cancer therapy that generates reactive oxygen species in tissues through photosensitive molecules under the irradiation of excitation light [153]. However, the therapeutic efficacy of PDT is significantly constrained by the oxygen content. It has been shown that in a low-oxygen environment, the amount of reactive oxygen species generated by PDT decreases significantly, and this change not only promotes the abnormal proliferation of tumor cells, but also induces irregularities in blood vessel growth, which in turn exacerbates the difficulty in the effective delivery of oxygenated blood and PDT drugs to the tumor site, thus constituting a major obstacle to the effectiveness of the therapy [154,155,156,157]. Therefore, cracking the problem of a hypoxic environment inside the tumor has become the key to breaking through the limitations of PDT treatment and enhancing its efficacy. Zeng et al. designed a bionic oxygen delivery system, BLICP@O_2_ (Figure 5) [158]. The system utilized hybrid tumor cell membranes and heat-sensitive liposomes as oxygen carriers and incorporated the NIR-II dye IR 1048, the photosensitizer dihydroporphyrinol e6 (Ce6), and perfluorohexane. Specific targeting was achieved by using tumor cell membrane-modified nanocarriers, which facilitated drug delivery to the tumor site [147,148], and simultaneously evaded phagocytosis by the leukocytes of the host effectively [159]. The experimental results showed that the photothermal effect triggered the controlled release of carried oxygen under 1064 nm laser irradiation. And 690 nm laser irradiation exhibited an enhanced PDT effect. Upon co-excitation by dual lasers (690/1064 nm), the system could precisely and rapidly release oxygen to improve the hypoxic microenvironment of HCC in order to enhance the efficacy of PDT.

### 3.3. Photothermal Therapy

**Figure 6:**
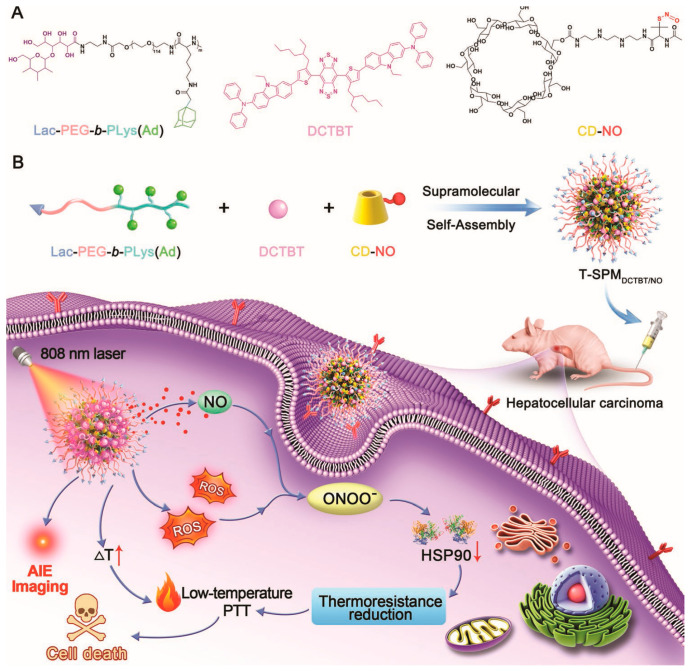
Supramolecular nanocarrier T-SPM_DCTBT/NO_ for ONOO^-^-enhanced mesophilic PTT in HCC. (**A**) The basic chemical structures of the components of nanocarrier. (**B**) Schematic illustration of the supramolecular nanocarrier T-SPM_DCTBT/NO_ for ONOO^-^-potentiated mild-temperature PTT of HCC [165]. Reprinted with permission from [165]. Copyright 2023, Wiley.

Photothermal therapy achieves tumor cell destruction by converting light energy into cytotoxic heat energy [160,161]. Conventional PTT requires high temperatures above 50 °C for tumor cell killing, which may cause severe damage to cells in adjacent normal tissues. Therefore, mesothermal PTT is a more promising anti-tumor strategy [162,163,164]. A novel supramolecular drug delivery system was designed by Hu et al. The system cleverly exploited the assembly of a lactose-modified poly(ethylene glycol)-poly(lysine) block copolymer (Lac-PEG-b-PLys(Ad)) with NO-releasing β-cyclodextrin derivatives (CD-NO) to form supramolecular polymer micelles through host–guest recognition (Figure 6) [165]. Lactose, as a targeting molecule, was specifically recognized and anchored to the overexpressed desialylated glycoprotein receptor of hepatocellular carcinoma cells, which achieves the precise targeting and delivery of the drug to HCC tissue [166,167]. When these drug-loaded nanocarriers are ingested by hepatocellular carcinoma cells, they are expected to trigger the release of NO under the environment of a high intracellular glutathione (GSH) concentration. Subsequently, NO can further react with ROS to generate the potent signaling molecule peroxynitrite (ONOO^-^) [168,169]. In those studies, the combination of nitric oxide (NO), which had NIR-II aggregation-induced emission (AIE) properties, and the photothermal agent DCTBT was designed to take advantage of the strong oxidative and nitrative potentials of the generated ONOO- to inhibit the expression of heat shock proteins (HSPs), which significantly reduced the heat resistance of the cancer cells and enhanced the therapeutic efficacy of the PTT to achieve a more effective and gentle tumor killing. This strategy not only broadened the application field of supramolecular drug delivery systems, but also provided a novel and efficient combination therapy for cancer treatment.

### 3.4. Immunotherapy

**Figure 7:**
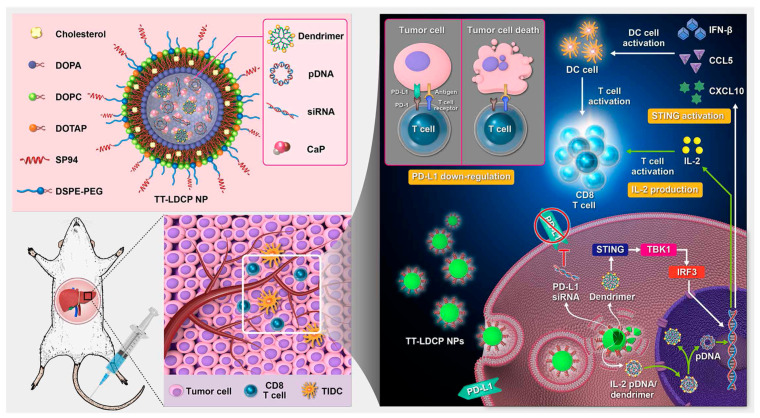
Mechanisms of immune–gene therapy with siRNA-containing TT-LDCP NPs against the immune checkpoint PD-L1 and pDNA encoding the immunostimulatory cytokine IL-2 [172]. Reprinted with permission from [172]. Copyright 2020, The American Association for the Advancement of Science.

Immunotherapy of liver tumors is particularly challenging due to the unique immune-related microenvironment of the liver, which makes it an organ of immune tolerance [170]. Activation of the interferon gene loop GMP-AMP synthase stimulator (cGAS-STING) pathway is an effective anti-cancer immunotherapy strategy for HCC [171]. Huang et al. applied this strategy to construct lipid–dendritic calcium phosphate nanoparticles (TT-LDCP NPs) that can be used as strong support for immunotherapy (Figure 7) [172]. TT-LDCP NPs contain siRNAs targeting the immune checkpoint ligand PD-L1 and plasmid DNA encoding the immunostimulatory factor IL-2, which can work together to inhibit immune-suppressive signals while enhancing immune-activating signals [173,174], which promotes tumor infiltration and CD8+ T-cell activation [175,176], and effectively curbs the progression of HCC. The presence of a tumor-targeting peptide (SP94) allowed the nanoparticles to specifically accumulate at the HCC tumor site [47,177], significantly enhancing the delivery efficiency of therapeutic pDNA/siRNA to the interior of cancer cells. Inside the cell, the calcium phosphate core was responsive to low pH environments and achieved endosomal escape, thereby efficiently releasing the nucleic acid material it carried. The external thymidine-modified polyamidoamine (PAMAM) dendrimer activated the stimulator of interferon genes (STING)-cyclic GMP-AMP synthase (cGAS) pathway, thereby activating the immune system [178]. At the same time, it promoted the escape of pDNA from endosomes and facilitated its entry into the nucleus for efficient gene transfection [179]. As a powerful adjuvant for immunotherapy, it stimulates the cellular immune response of the body. Together with this series of well-designed mechanisms, the nano vaccine opened up a new way for immunotherapy of hepatocellular carcinoma and shows a broad application prospect.

### 3.5. Collaborative Treatment

In the treatment of HCC, it is often difficult to achieve the desired results using only a single treatment. Therefore, researchers are currently working on the development of drug delivery systems that can efficiently synergize multiple therapeutic approaches. By targeting nanocarriers that integrate multiple therapeutic components to the target site, the therapeutic effect of HCC can be significantly improved, bringing more effective treatment options to patients. Ou et al. prepared ION-AAV 2 using the coupling of ION-carboxy and AAV 2-amines [180]. Adeno-associated virus (AAV), as an extremely efficient gene delivery vector, has demonstrated its remarkable capabilities in the field of gene therapy, especially in mediating gene expression, achieving gene silencing, and performing gene editing in several preclinical and clinical trials, which have resulted in significant breakthroughs [181,182]. Currently, the application of AAV has been successfully extended to the clinical treatment of HCC, enabling liver targeting [182]. Negatively charged ION downregulates M2-associated arginase-1 in macrophages. Upregulating the interferon regulatory factor 5 signaling pathway led to the polarization of M1-type macrophages [183]. Anti-tumor M1 macrophages significantly promote CD8^+^ T-cell infiltration [184,185]. ION-AAV 2 produced both immunotherapeutic and photodynamic virotherapy for HCC. KillerRed is a low-immunogenic green-like fluorescent protein dimer containing a chromophore (Gln 65-Tyr 66-Gly 67) in its β-barrel structure, which efficiently absorbs green color in the range of 540–580 nm, and emits longer wavelengths of red light at 610 nm [186,187,188,189]. This light-activated mechanism promoted the production of reactive oxygen species (ROS) by exchanging oxygen and ions with the adjacent environment. When tumor cells were infected with AAV 2–KillerRed, the KillerRed photosensitizer was expressed in the cells and can be activated by light to initiate photodynamic virotherapy and promote cell death. At the same time, ION increased the ratio of M1/M2 type macrophages and enhanced their immunomodulatory function. The combination of the two mechanisms ultimately led to a reduction in tumor size.

**Figure 8:**
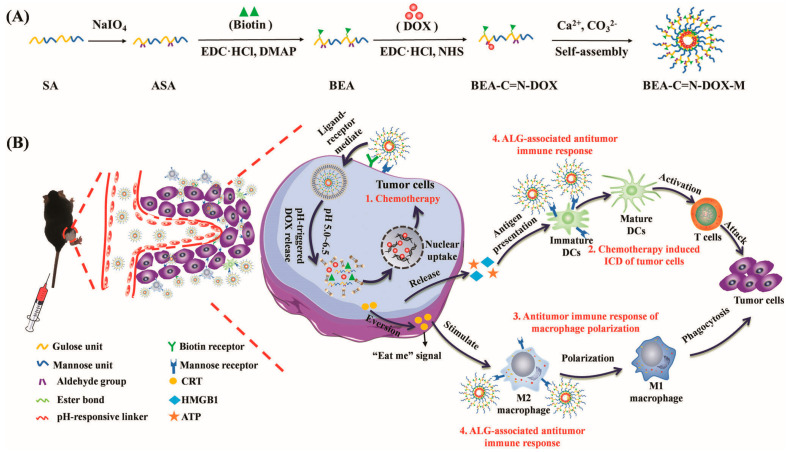
Schematic diagram of BEA-C=N-DOX-M for synergistic chemoimmunotherapy of hepatocellular carcinoma. (**A**) The synthesis process of BEA-C=N-DOX-M. (**B**) The action process of this nano-micelles in vivo and the strategy for achieving chemo-immunotherapy in HCC [190]. Reprinted with permission from [190]. Copyright 2023, Wiley.

Biotinylated aldehyde–alginate–adriamycin nano-micelles (BEA-C=N-DOX-M) with supreme stability in saline at the physiological pH of 7.40 were developed by Huang et al. (Figure 8) [190]. Specifically, Bio (a vitamin with high tumor-targeting specificity) and DOX bind to oxidized alginate via ester- and acid-sensitive imine bonds, respectively, and were used as targeting and therapeutic moieties for synergistic chemoimmunotherapy [191]. Alginic acid contains β-D-mannuronic acid and α-L-guluronic acid [192], of which β-D-mannuronic acid could have ligand–receptor interactions with MR and deliver drugs to tumor cells via MR, and α-L-guluronic acid could be coupled with DOX. The nano-micelles combined the natural immune regulatory function of alginate with the chemotherapeutic function of DOX, while demonstrating drug-targeting to HCC cells through drug interaction with MRs and biotin-mediated endocytosis.

## 4. Conclusions and Outlook

HCC is a complex pathological process that involves multiple stages, and its occurrence and progression are deeply regulated by many factors, which greatly increases the difficulty of developing effective therapeutic interventions for this disease. Currently, surgical resection and liver transplantation are the established treatments for patients with advanced HCC, but their limitations cannot be ignored. The rise of nanomedicines and immunotherapies has brought new hopes and possibilities for the successful treatment of HCC, which indicates that these emerging therapies may become a key force in changing the treatment landscape of HCC. Combining the characteristics of the unique microenvironment of hepatocellular carcinoma, the functionalized design of nanoparticles can not only enhance their responsiveness at the tumor site, but also significantly improve their targeting precision. This design idea aims to enable the nanomedicine system to penetrate the tumor tissue more efficiently and act precisely on the cancer cells, thus achieving a more precise and effective treatment for liver cancer.

However, the application of nanotechnology for the targeting of HCC still has some unresolved issues that require further attention from researchers.

(1) Currently, the therapeutic effect of a single treatment modality for HCC is limited. How to innovatively develop a synergistic strategy between multiple therapeutic means, so that the therapeutic effects of various therapeutic mechanisms can be mutually enhanced and they can complement each other’s strengths, and together play a more significant tumor suppression, has become a direction of exploration for subsequent studies.
(2) The biosafety of nanomedicines cannot be guaranteed due to the lack of a unified and standardized methodology for biosafety evaluation in current research [28]. For example, heavy metal components may potentially impair liver and kidney function, while the acidic and alkaline nature of nanoparticles may cause damage to the walls of blood vessels, in addition to the fact that numerous nanomaterials may trigger significant changes in hemodynamics, all of which may further contribute to the failure of vital organs of the body. Regulators lack a clear or even harmonized regulatory assessment pathway for complex medicines such as nanomedicines. In the EMA’s evaluation process, in addition to routine human bioequivalence or pharmacokinetic (PK) data, non-clinical evaluations need to be supplemented to add valuable and comparable data through a stepwise approach. In contrast, the FDA does not rely on non-clinical evaluations of such nanoparticle drugs because they believe that animal data may not accurately predict the human response [193]. Therefore, there is a need to explore and assess the biological safety of drugs, including, but not limited to, toxic responses, in vivo distribution, metabolic pathways, and long-term effects. By establishing a more comprehensive, scientific, and standardized biosafety evaluation system, safer and less toxic nanomedicines that are truly beneficial to the human body can be screened for.
(3) Although short-term drug therapy may provide some relief from the symptoms of HCC, HCC remains a disease with a relatively poor prognosis. Particularly in advanced HCC cases and those who are multidrug-resistant, the development and implementation of treatment regimens face significant challenges [194]. The mechanism of multidrug resistance (MDR) in HCC is intricate and complex, and how to overcome this problem and improve the prognosis of hepatocellular carcinoma patients remains the future direction of nanomedicine.

In general, the development of nanotechnology offers broad prospects for the treatment of HCC. Although researchers still need to face and solve a series of complex problems in this process, these challenges cannot hide the great value that nanomedicine has shown in the field of cancer diagnosis and treatment. As research continues to deepen and technology continues to advance, we are fully confident that nanomedicine will occupy an indispensable and important position in the future cancer treatment system. Its unique nanoscale advantages not only provide a more precise and efficient way of drug delivery, but also bring unprecedented opportunities for personalized medicine and improved treatment outcomes and quality of life for patients. Therefore, despite the challenges ahead, the future of nanomedicine remains promising.

## Figures and Tables

**Table 1:** Summary of advanced HCC treatments with positive results in phase III clinical trials [22].

Agent	Target	Clinical Stage
Sorafenib	VEGFR1–3, PDGFR, RAF, KIT	FDA-approved (2007)
Regorafenib	VEGFR1–3, PDGFR, RAF, FGFR1–2	FDA-approved (2017)
Lenvatinib	VEGFR1–3, PDGFR, FGFR1–4, RET	FDA-approved (2018)
Pembrolizumab	PD1	FDA-approved (2018)
Cabozantinib	VEGFR1–3, MET, RET	FDA-approved (2019)
Ramucirumab	VEGFR2	FDA-approved (2019)
Atezolizumab + bevacizumab	PDL1 + VEGFA	FDA-approved (2020)
Donafenib	VEGFR1–3, PDGFR, RAF	Phase III (2020)
Apatinib	VEGFR2, KIT, RET, SRC	Phase III (2021)
Sintilimab + IBI305	PD1 + VEGFA	Phase III (2021)
Atezolizumab + cabozantinib	PDL1 + multiple targets	Phase III (2021)
Durvalumab + tremelimumab	PDL1 + CTLA4	Phase III (2022)
Durvalumab	PDL1	Phase III (2022)

## Data Availability

Data are available on request.
